# Awareness of Female Genital Mutilation/Cutting Among the General Population in 2019: A Survey-based Study in Saudi Arabia

**DOI:** 10.7759/cureus.6651

**Published:** 2020-01-14

**Authors:** Mohammed Malak, Duaa Basalem, Shahad Aleiidi, Nashwa Helabi, Meaad Almutairi, Alya Alhamed

**Affiliations:** 1 Obstetrics and Gynecology, Faculty of Medicine, King Abdulaziz University, Jeddah, SAU; 2 Radiology, Faculty of Medicine, King Abdulaziz University Hospital, Jeddah, SAU

**Keywords:** female genital mutilation, female genital cutting, female circumcision, saudi arabia, obstetrics and gynecology, urogynecology, public awareness

## Abstract

Background

Female genital mutilation/cutting (FGM/C) includes “all procedures that include the removal of the external female genitalia, partially or totally, using a blade or shard of glass for non-medical reasons.” FGM/C is a harmful procedure known to violates a person's human rights. This study aims to assess the level of knowledge regarding FGM/C in Saudi Arabia.

Methodology

A survey-based cross-sectional study was conducted in 2019 among a representative sample of the general population of Saudi Arabia. The questionnaire consisted of a set of questions dealing with the knowledge of FGM/C. We analyzed the data by using the Statistical Package for Social Sciences (SPSS) version 21 IBM Corp., Armonk, NY), and the results were acquired by applying Pearson’s chi-square test and calculating the frequencies.

Results

The study included 659 participants; 80.4% were females and 19.6% were males. About 93.8% knew about FGM/C while 68.3% were aware of the health consequences of female circumcision. The most common sources of information were the Internet and social media (54.4%). The overall prevalence of circumcision among the female participants was 9.4%.

Conclusion

Generally, our population had good knowledge of FGM/C. However, it is not enough to eliminate this practice. Health and human rights education campaigns and programs should be done to enhance public awareness. In addition, more researches about the prevalence of this tradition should be taken into consideration.

## Introduction

The World Health Organization (WHO) defines female genital mutilation/cutting (FGM/C) as “all procedures that include removal of the external female genitalia partially or totally, using a blade or shard of glass for non-medical reasons.” FGM/C is mostly performed in girls between infancy and the age of 15 [1].

The exact prevalence of females worldwide who have undergone FGM/C is still unknown. However, more than 200 million females had undergone this procedure in 30 countries in Africa, the Middle East, and Asia where FGM/C is concentrated [2].

There are four major types of FGM/C procedures, ranging from partial or complete removal of the clitoris to infibulation, where the vaginal opening is narrowed by manipulation of the inner or outer labia and sometimes through stitching [3-4]. FGM/C is a harmful procedure with no health benefits for women and is well-known as a procedure that violates a person's human rights, as well as increases their risk for health complications [5]. These complications include hemorrhage, severe pain, infections, and serious psychological trauma. Long-term complications include urinary problems, obstetrical complications, including emergency cesarean section, variable degrees of vaginal lacerations, and ongoing sexual and psychological issues [3,6-11].

Many studies were conducted regarding this issue worldwide. For example, a study done in Nigeria in 2007 reported that out of 260 of the population, 94.6% knew about FGM/C. Most of them (83.8%) wanted the procedure to be suspended. However, nearly 14.6% wanted to circumcise their daughters [12].

Another cross-sectional study was conducted in Egypt, in 2013, among 600 medical students. In this, 14.7% of female students were circumcised and 40% reported to have adverse consequences while around 46.8% were supporting the discontinuation of this practice [13]. A study conducted in Egypt in 2016 to assess the level of knowledge and beliefs about FGM/C revealed that out of 618 participants, 56% support the continuation of the practice. FGM was performed on 76.6% of the female respondents, and 35.6% of them experienced complications [14]. In 2016, a study was conducted in Ombada Province, Khartoum State, Sudan, about awareness of female circumcision among mothers. Out of the 368 respondents analyzed, 80.2% confirmed that FGM/C is still practiced. The purposes behind the persistence of this practice are to Ensure virginity (52.2%), follow religious guidance (32.9%), avoid social discrimination (10.2%), and good for prospective marriages (4.7%) [15]. In Saudi Arabia, a study in 2007 revealed that sexual function in women with FGM is unfavorably affected [16]. Another study conducted in King Abdulaziz University Hospital, Jeddah, Saudi Arabia, in 2010 reported that a clitoral cyst is a common complication of FGM/C, where 15 women (46.9%) out of 32 females who underwent surgical excision of epidermal clitoral cysts had a previous history of FGM/C [17].

Since FGM/C is still widely practiced in our society, regardless of its well-known consequences, the community's awareness and knowledge regarding this topic are significantly important. Although there are multiple studies conducted about the complications of FGM/C in Saudi Arabia, unfortunately, few were addressing the awareness and beliefs of this procedure. Our aim of conducting this study is to assess the level of awareness of FGM/C among the general population of Saudi Arabia.

## Materials and methods

This is an observational cross-sectional study that was approved by the institutional review board (IRB) of KAUH, Jeddah, Saudi Arabia. A total of 659 participants from the general population of Saudi Arabia in 2019 were asked to complete an electronic, self-administered questionnaire to assess their knowledge of FGM/C. We included all Saudi Arabia residents aged 18 years and older.

The questionnaire was validated by a panel of obstetrics and gynecology experts. It consisted of 19 questions divided into two sections. The first one contained the demographic data of participants such as age, gender, nationality, region, marital status, and educational level. The second section evaluated the knowledge about FGM/C reasons, consequences, practitioners, and preventive measures. Consent was taken from all the participants before completing the questionnaire.

The collected data was arranged in Google forms and entered through the Statistical Package for Social Sciences (SPSS) 21 (IBM Corp., Armonk, NY). The analysis was done through different statistical tests, such as frequency tables and chi-square tests, to evaluate the level of knowledge of Jeddah residents regarding FGM/C, and a p-value < 0.05 was considered significant.

## Results

In this study, we aim to estimate the level of knowledge of FGM/C among the general population of Saudi Arabia. The study included 659 participants; 80.4% were females and 19.6% were males. The most frequent age category was 18-30 accounting for 78.9%. The level of education for most of the participants was a bachelor’s degree (63.1%). Other demographic data were collected as shown in Table 1.

**Table 1:** Demographic data of the 659 participants included in the study

Demographics		Participants [N (%)]
Gender	Female	530 (80.4)
	Male	129 (19.6)
Age	18 - 30	520 (78.9)
	31 - 40	98 (14.9)
	41 - 50	22 (3.3)
	51 - 60	14 (2.1)
	61 and above	5 (0.8)
Level of education	Primary school	1 (0.2)
	Intermediate school	2 (0.3)
	Secondary school	139 (21.1)
	Diploma	40 (6.1)
	Bachelor	416 (63.1)
	Higher	61 (9.3)
Involved in the medical field	Yes	176 (26.7)
	No	483 (73.3)
Marital status	Single	450 (68.3)
	Married	195 (29.6)
	Divorced	12 (0.8)
	Widow	2 (0.3)
Nationality	Saudi	591 (89.7)
	Non-Saudi	68 (10.3)
Saudi regions	West Region	201 (34.0)
	East Region	71 (12.0)
	North Region	31 (5.2)
	South Region	60 (10.2)
	Central Region	228 (38.6)

**Figure 1:**
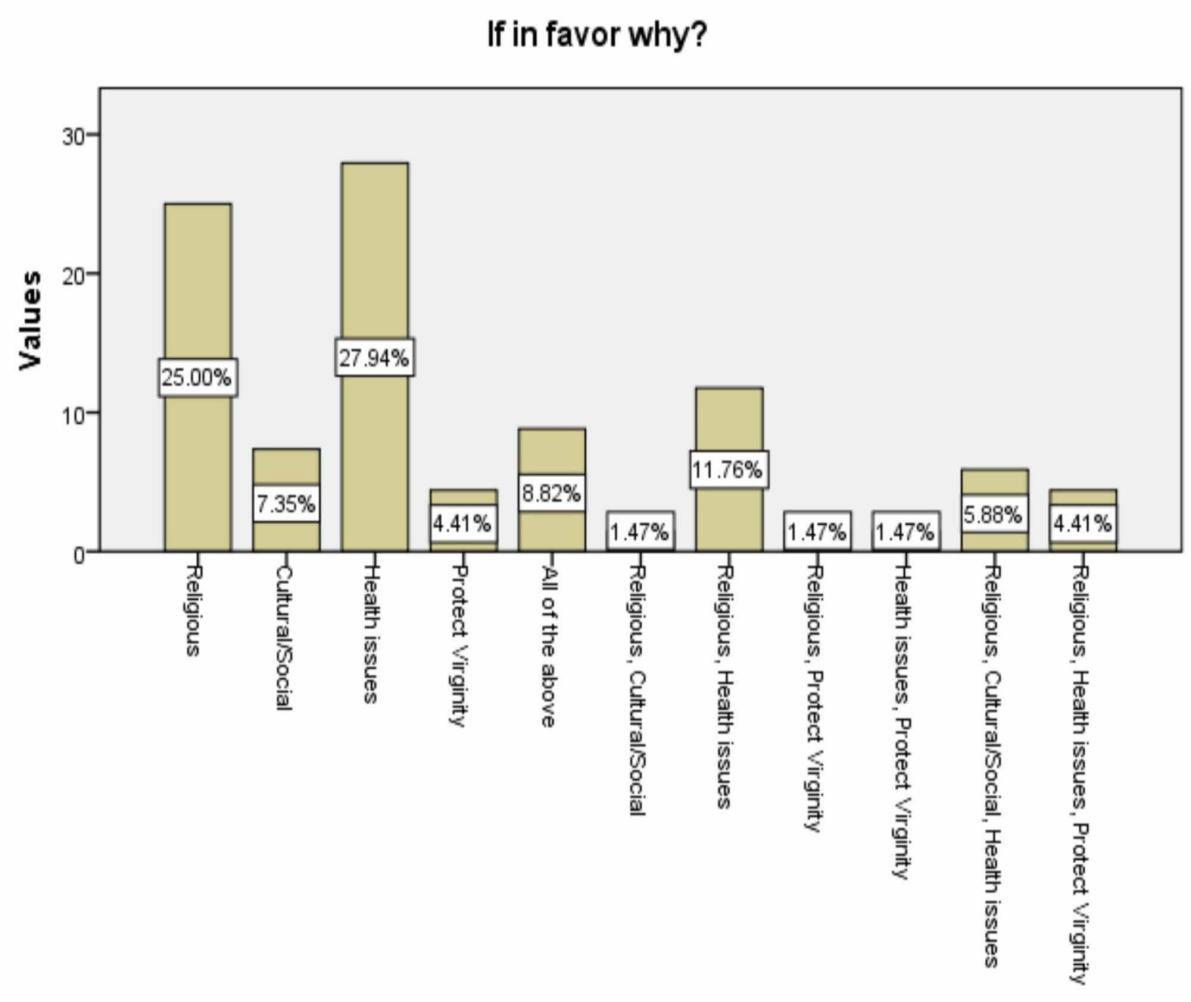
Supporters reasons

The majority of the participants were aware of FGM/C, accounting for 93.8%. Ten point three percent were in favor of female circumcision, citing hygiene, religion, tradition, and preservation of virginity as their reasons (Figure 1).

**Figure 2:**
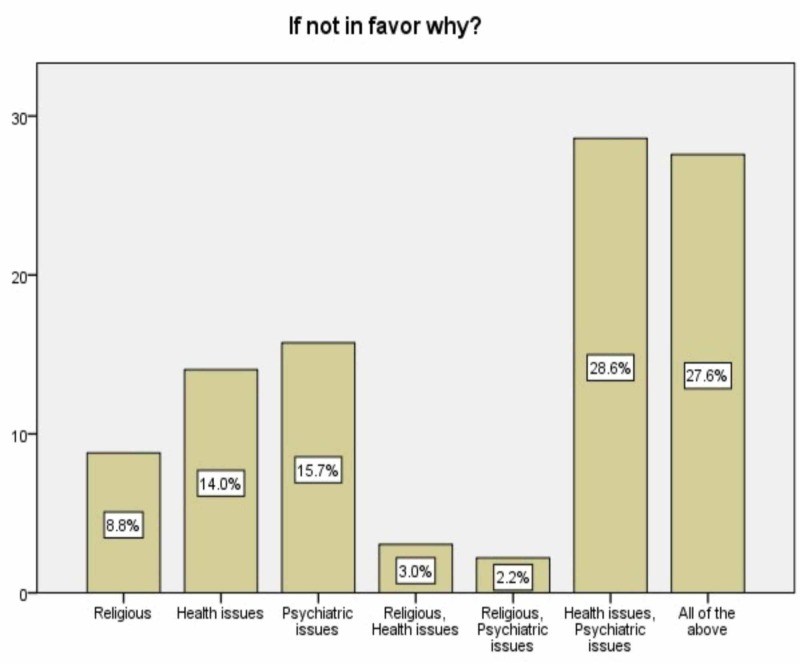
Opponents reasons

However, most of the respondents (89.7%) were against this practice (their reasons are shown in Figure 2).

**Figure 3:**
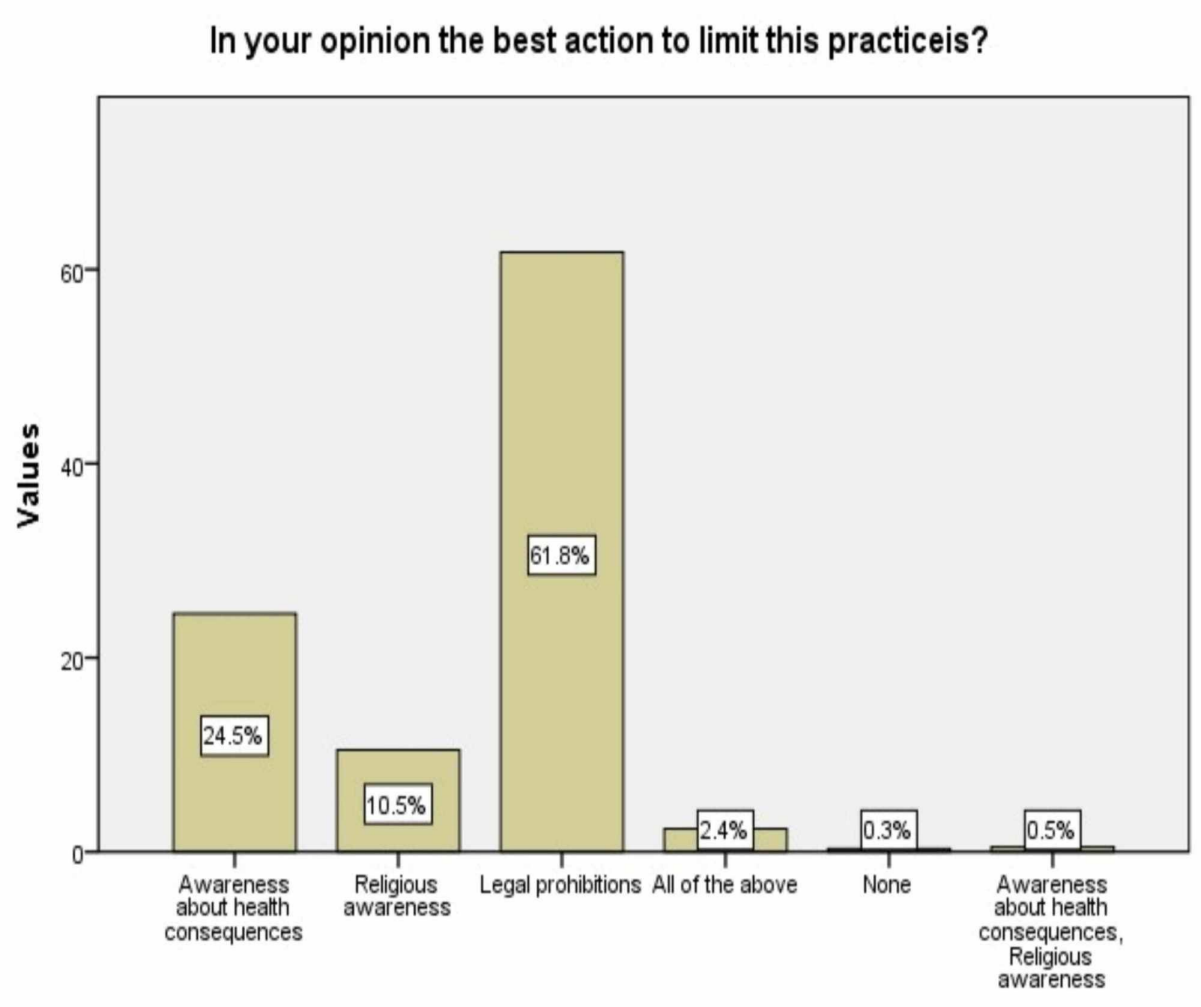
Ways to limit FGM/C FGM/C: female genital mutilation/cutting

Sixty-one point eight percent of the opponents chose legal prohibition as the best action to limit this practice (Figure 3).

68.3% knew about the health consequences of female circumcision. The most recognized one was psychiatric issues, accounting for 86.7% (Table 2).

**Table 2:** Health consequences of FGM/C FGM/C: female genital mutilation/cutting

	Agree	Disagree	I don't know
Severe pain	80.4%	1.3%	18.2%
Bleeding	75.8%	1.6%	22.7%
Inflammations of genitalia	84.2%	1.3%	14.4%
Urination difficulties	67.1%	6.4%	26.4%
Obstetric complications	52.4%	8.2%	39.3%
Decreased/Loss of libido	84.7%	2.2%	13.1%
Dyspareunia	56.4%	6.4%	37.1%
Dysmenorrhea	42.2%	10.0%	47.8%
Psychiatric issues	86.7%	2.7%	10.7%

**Figure 4:**
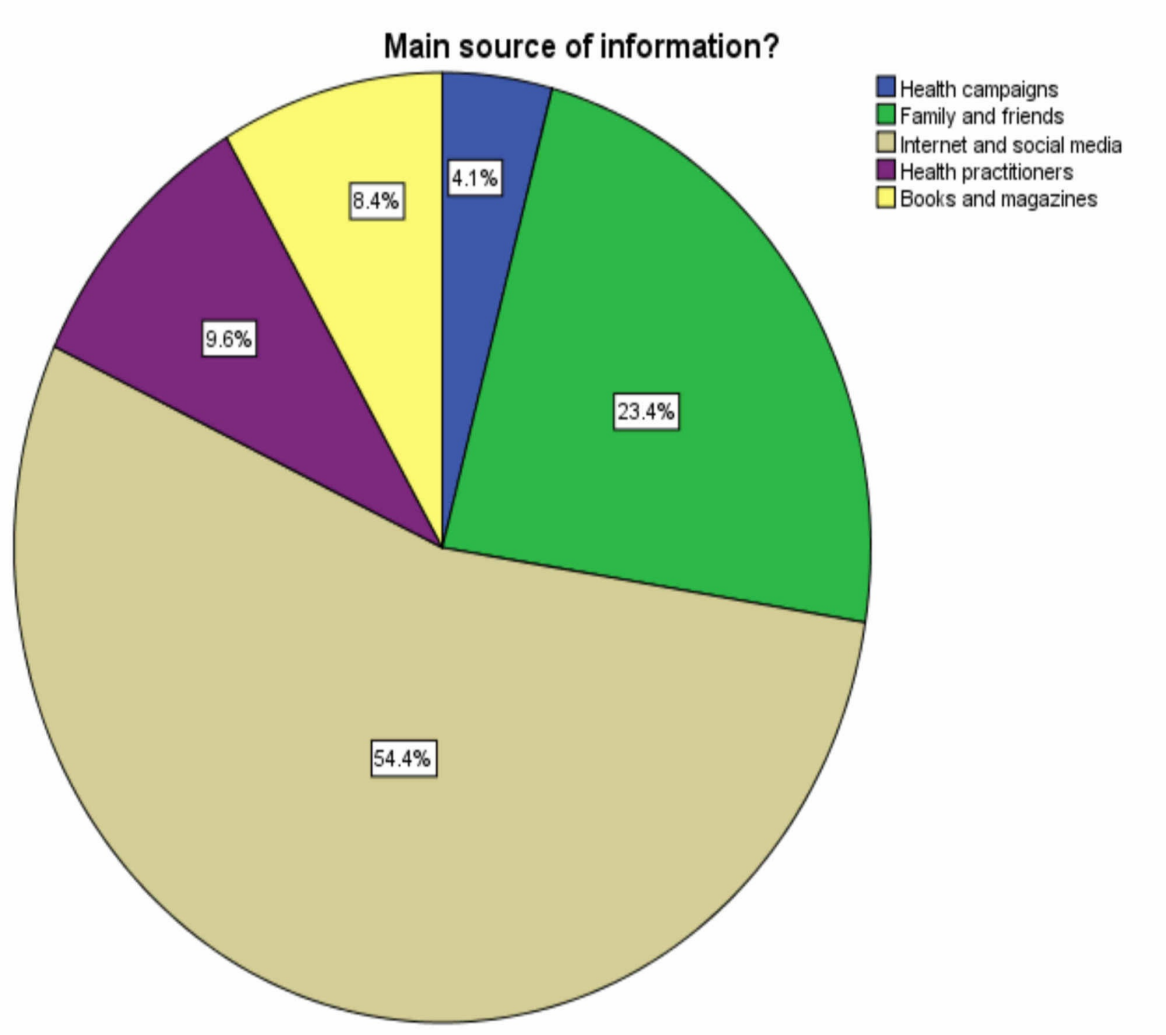
Source of information

Regarding the practitioners of FGM/C, the majority of the respondents (49.3%) chose traditional practitioners as the most involved in this practice. Other options were health practitioners in hospitals or primary health care centers, accounting for 7.3% and 4.7%, respectively. The remaining had no information. The most common sources of information chosen by the participants were the Internet and social media (54.4%), followed by family and friends (23.4%), as shown in Figure 4.

**Figure 5:**
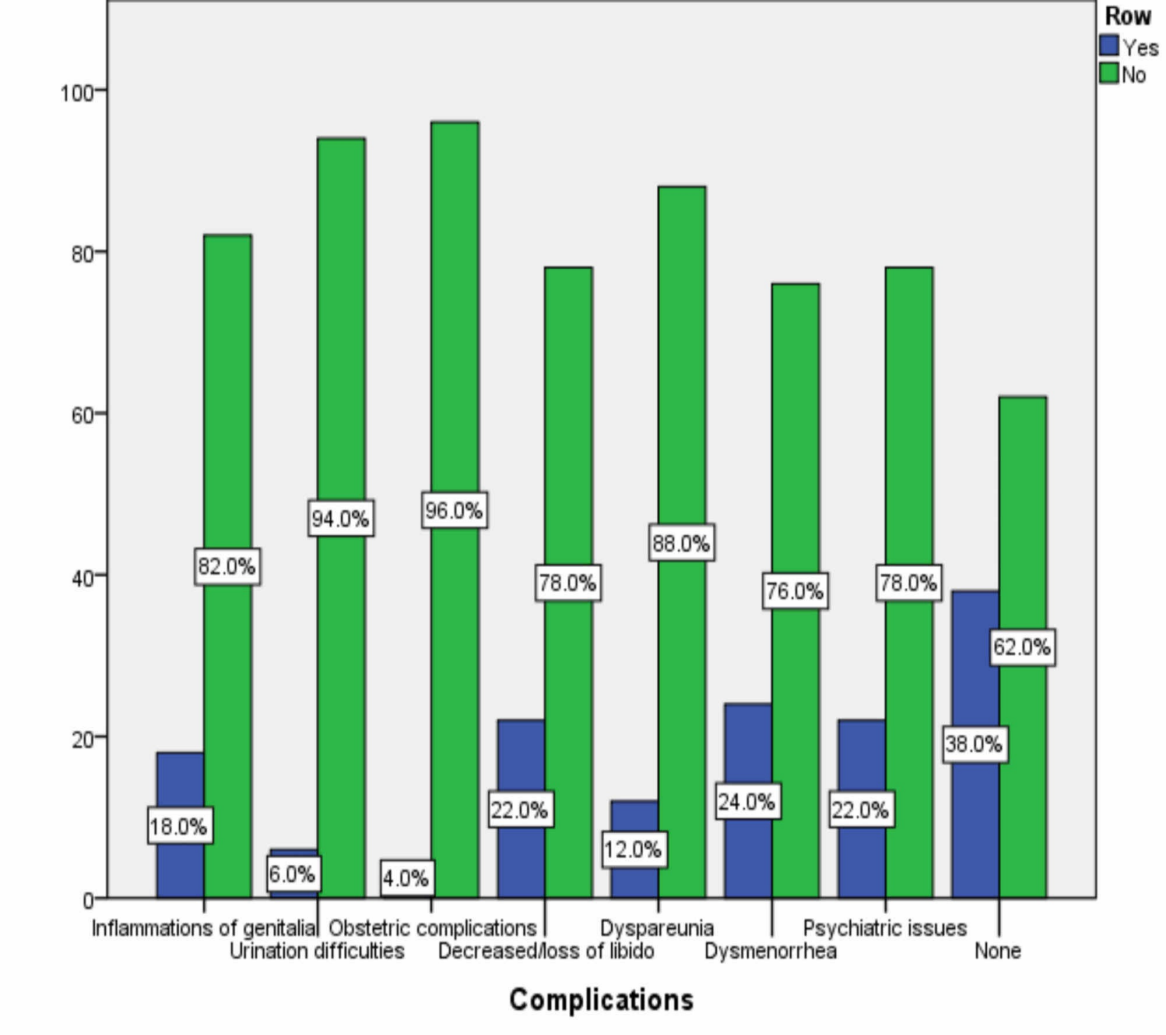
Complications of FGM/C FGM/C: female genital mutilation/cutting

The overall prevalence of circumcision among the female participants was 9.4%. Most of them were circumcised at the age of one day to a month (52.0%). Other age categories were two months to a year (18.0%), two to five years (6.0%), and six to 15 years (10.0%). Fourteen percent of them didn’t remember. Regarding the health consequences, dysmenorrhea was the most chosen by them (Figure 5). However, 38.0% provided that they didn’t experience any health consequences.

Significant associations were found between the nationality and favor of FGM/C, awareness of the health consequences, involved practitioners, and history of circumcision (p-value= 0.000). In addition, positive relationships were established between the different regions of Saudi Arabia and being familiar with female circumcision, the favor of FGM/C, awareness of the health consequences, involved practitioners, and history of circumcision, as demonstrated in Table 3.

**Table 3:** Cross-tabs (chi-square) between the Saudi regions and familiarity with FGM/C, awareness of health consequences, involved practitioners, and history of circumcision FGM/C: female genital mutilation/cutting

Variable	P-value
Heard about FGM/C	0.008
Favor of FGM/C	0.013
Awareness of health consequences of FGM/C	0.049
Involved practitioners	0.000
History of circumcision	0.000

Significant associations were established between the source of information and awareness of the health consequences, the favor of FGM/C, involved practitioners, and other variables, as shown in Table 4. Additionally, the favor of FGM/C was found to be significantly associated with gender (p-value= 0.000).

**Table 4:** Cross-tabs (chi-square) between the source of information and awareness of the health consequences, favor of FGM/C, and involved practitioners FGM/C: female genital mutilation/cutting

Variable		P-value
Favor of FGM/C		0.000
If in favor, why?		0.001
If not in favor, why?		0.000
The best action to limit this practice		0.001
Awareness of health consequences of FGM/C		0.000
Health consequences	Severe pain	0.000
	Bleeding	
	Inflammation of the genitalia	
	Urination difficulty	
	Obstetric complications	
	Decreased/loss of libido	
	Dyspareunia	
	Dysmenorrhea	
	Psychiatric issues	
Involved practitioners		0.000

## Discussion

This is a community-based, cross-sectional study that aims to evaluate the level of awareness of FGM/C among the residents of Saudi Arabia.

Our results show that a high percentage of the participants had heard about female circumcision. A similar result was found in studies conducted in Sudan and Nigeria in which 100.0% and 90.5% were familiar with the term, respectively [15,18]. This could be explained by the fact that female circumcision is still widely practiced in many countries around the world, particularly Africa, the Middle East, and Asia [2].

Regarding the supporters of FGM/C, the highest percentage cited health benefits, particularly hygiene, as the main reason. Hygiene was also established as one of the contributing factors of this practice in a systematic review conducted in 2013 [19]. Religion came second among the influential factors, followed by culture, tradition, and insuring virginity. These factors were identified in many previous studies. For example, Esmeal EA et al. reported that the preservation of virginity, following religious instructions, and cultural reasons were significant motivations for the continuation of FGM/C practice [15]. An explanation of this is that FGM/C is deeply rooted in many cultures and societies through multiple links to tradition, religion, safeguarding virginity, and moral standards [20].

According to our data, the majority of the participants were against female circumcision citing health issues, whether it be psychological or physical, as their main reason. Similarly, health issues were reported by many other studies as one of the most prominent factors of female circumcision opposition [19].

Religion was another reason, chosen by 41.7% of the opponents. This could be attributed to the different arguments among the Islamic law schools regarding this practice, where it’s considered obligatory in the Shafi’i law school, and only recommended in the other law schools [19].

Regarding FGM/C eradication, most of the opponents chose legal prohibition as the best action to limit this practice, followed by raising awareness about the health consequences of this practice. Laws have shown to be effective in slowing down the continuation of this ritual. A systematic review conducted in this field reported that informing the citizens continuously that FGM/C is a violation of the law and human rights are necessary [19].

With regard to the role of health education and awareness in the eradication of FGM/C, similar results were found in Esmeal EA et al. and Ibekwe et al., as both of these studies revealed that awareness campaigns and health education were suggested by the participants as the best actions in the elimination of this ritual [15,18].

Our study shows that the majority of participants were aware of the health consequences of female circumcision; this finding is similar to the results of the study conducted in Sudan and Egypt in which 75% and 57.9% knew about the health consequences of female circumcision, respectively [14-15]. Psychiatric issues was the most chosen one by the participants in our study. However, a study conducted in Sudan, bleeding and urination difficulties were the most known by the participants. However, their research was done on a smaller sample size (368), and it was targeting only the mothers, therefore, making a direct comparison is difficult [15]. Regarding practitioners of female circumcision, the most identified category was traditional practitioners. Similar results were reported by studies conducted in the rural Gambia and in Sudan [15,21].

According to our result, most participants were circumcised below the age of one year (70%); a similar study from Nigeria showed that 88.2% of women were circumcised before the age of one year [22]. However, in other studies, there were different age groups at which most of the girls have been circumcised. In a study from Iraq Kurdistan, most of the participants chose four to seven years as the age at which they mutilated [23]. Another study from Eastern Ethiopia reported that 11-12 years is the most common age group [24]. This difference in the results could be owed to the variations in each country, ethnicity, and religion [23].

Regarding the health consequences of FGM, most mutilated females reported that they didn’t have any complications; this could be explained by the fact that most of the women did not correlate the complication to FGM or they didn’t remember the experience [23].

In our study, we found a significant relationship between nationality and multiple variables, as mentioned above. An explanation for this could be the direct association between the prevalence of FGM/C among certain nationalities and the mentioned variables. An example of this relationship is Sudan, where a study conducted there revealed that the prevalence of FGM/C was 80.2% and the level of awareness of this practice was 100% [15].

We find that positive relationships were established between the different regions of Saudi Arabia and other variables. However, the deficiency of studies of FGM/C in Saudi Arabia affects the effectiveness of such a comparison to explain this relation.

The present study found a significant association between the source of information and many different variables demonstrated in Table 4. This confirms the importance of employing all possible sources to raise awareness and correct the misconceptions regarding this practice.

Our study shows that the majority of our participants were against this practice; most of them were females. A similar result in a study conducted in Egypt among medical students found that the discontinuation of FGM/C was favored by most of the students (58.7%) especially females. An explanation for this could be due to the lack of female reproductive health education programs in the male's educational curriculum [13].

Although this study was carefully conducted, some limitations were unavoidable. A similar percentage of gender, age, regions, and educational levels could not be reached due to the random distribution of the questionnaire. This may have affected our results. However, such limitations are not always avoidable.

## Conclusions

The present study has estimated the level of awareness of FGM/C among the general population of Saudi Arabia. Our results showed that the majority of the participants have heard about female circumcision. Most of the participants tend to get their information about this practice from the Internet and social media. For these reasons, healthcare professionals need to work together to raise public awareness through every means possible, from educational campaigns and programs to social media platforms, to help raise awareness and correct misunderstandings regarding this ritual. Moreover, future research in this area is needed to evaluate the prevalence of FGM/C, particularly among the different regions of Saudi Arabia.
